# Diagnostic value of MRI in traumatic triangular fibrocartilage complex injuries: a retrospective study

**DOI:** 10.1186/s12891-023-07140-z

**Published:** 2024-01-13

**Authors:** Xuanyu Zhao, Aiping Yu, Huali Zhao, Yanqun Qiu

**Affiliations:** 1 Department of Hand and Upper Extremity Surgery, Jing’ an District Central Hospital, Fudan University, Shanghai, 200040 China; 2 Department of Plastic and Reconstructive Surgery, Shanghai Jiaotong University School of Medicine Affiliated 9th People’s Hospital, Shanghai, 200011 China; 3 Department of Radiology, Jing’an District Central Hospital, Fudan University, Shanghai, 200040 China; 4 National Clinical Research Center for Aging and Medicine, Fudan University, Shanghai, 200040 China; 5 Research unit of synergistic reconstruction of upper and lower limbs after brain injury, Chinese Academy of Medical Sciences, Shanghai, 200040 China; 6 Department of Hand Surgery, Huashan Hospital, Fudan University, Shanghai, 200040 China

**Keywords:** Arthroscopy, Diagnostic value, MRI, TFCC, Wrist

## Abstract

**Background:**

Triangular fibrocartilage complex (TFCC) injuries commonly manifest as ulnar-sided wrist pain and can be associated with distal radioulnar joint (DRUJ) instability and subsequent wrist functional decline. This study aimed to assess the diagnostic value of MRI compared to wrist arthroscopy in identifying traumatic TFCC injuries and to determine the distribution of different TFCC injury subtypes in a normal clinical setting.

**Methods:**

The data of 193 patients who underwent both preoperative wrist MRI and wrist arthroscopy were retrospectively reviewed. The analysis focused on the proportion of subtypes and the diagnostic value of MRI in traumatic TFCC injuries, utilizing Palmer’s and Atzei’s classification with wrist arthroscopy considered as the gold standard.

**Results:**

The most prevalent subtype of TFCC injuries were peripheral injuries (Palmer 1B, 67.9%), followed by combined injuries (Palmer 1 A + 1B, 14%; Palmer 1B + 1D, 8.3%). Compared with wrist arthroscopy, the diagnostic sensitivity, specificity, negative predictive value (NPV), and Kappa value of MRI was as follows: traumatic TFCC tears 0.99 (95% CI: 0.97-1), 0.90 (0.78-0.96), 0.97 (0.87-1), and 0.93; styloid lamina tears 0.93 (0.88-0.96), 0.53 (0.30-0.75), 0.47 (0.26-0.69), and 0.44; and foveal lamina tears 0.85 (0.74-0.92), 0.38 (0.29-0.49), 0.79 (0.65-0.89), and 0.21.

**Conclusions:**

The diagnostic value of MRI in traumatic TFCC injuries has been confirmed to be almost perfect using Palmer’s classification. In more detailed classification of TFCC injuries, such as pc-TFCC tears classified by Atzei’s classification, the diagnostic accuracy of MRI remains lower compared to wrist arthroscopy. Radiological associated injuries may offer additional diagnostic value in cases with diagnostic uncertainty.

## Background

The triangular fibrocartilage complex (TFCC) is a fibrocartilaginous structure located between the medial surface of the distal radius and the ulnar head, comprising the articular disc, the meniscus homologue, the dorsal and palmar radioulnar ligaments, the ulnolunate and ulnotriquetral ligaments, and the extensor carpi ulnaris tendon sheath [1, 2]. TFCC functions as the primary stabilizer of the ulnar aspect of the wrist [3]. Injuries of TFCC commonly occur following a fall on the upper limb or a rotational forearm injury. They often manifest as ulnar-sided wrist pain and are frequently associated with distal radioulnar joint (DRUJ) instability, leading to functional decline [4]. Degenerative alterations are also a main pathogenic factor of TFCC injuries, usually associated with age changes, prolonged labor work, and ulnar positive variance [5, 6].

According to Palmer’s classification, TFCC injuries can be subtyped into traumatic (type 1) or degenerative (type 2) injuries [7]. However, this classification is unable to cover all kinds of TFCC injuries. For instance, volar and dorsal TFCC injuries were not categorized, and Palmer’s classification does not encompass frequently encountered combined injuries observed in clinical practice [1, 8, 9]. Moreover, in 2009, Atzei introduced a treatment-oriented classification of TFCC peripheral injuries, in virtue of advances in radiocarpal and DRUJ diagnostic arthroscopy [3]. In 2022, Schmitt et al. introduced a novel “CUP” classification system designed specifically for TFCC lesions, but it does not consider the adjacent distal radioulnar joint and ulnar carpus [10]. To our knowledge, there’s limited research on the proportion of different types of TFCC injuries or the prevalence of various types of combined injuries.

Physical, radiological, and clinical examinations are all valuable for determining the diagnosis of TFCC injuries. Physical examinations such as the “ulnar fovea sign” and “ulnar grinding test” can assist in diagnosing TFCC injuries, but there is a need to enhance their specificity and sensitivity [8, 11, 12]. MRI scan is commonly utilized as diagnostic tool for evaluating TFCC due to its non-invasiveness, accessibility, and spatial resolution [13, 14]. But MRI scan may not be able to accurately assess the size and location of TFCC injuries [15]. As the “gold standard” for identifying TFCC injuries, wrist arthroscopy is the only diagnostic tool that dynamically can assess the grade of instability and the healing capacity of the injury, serving both for diagnosis and repair of the injured TFCC [16–18].

Therefore, the objectives of this study are to evaluate the value of MRI in the qualitative diagnosis and localization of traumatic (Palmer type 1) TFCC injuries compared with arthroscopy in a normal clinical setting, and to determine the distribution of different subtypes of TFCC injuries at our center.

## Methods

### Patients

This retrospective study included a total of 193 consecutive patients from July 2020 to May 2022. The inclusion criteria were as follows: explicit trauma history, preoperative MRI, underwent wrist arthroscopy, arthroscopically verified traumatic TFCC injuries, and that time interval between preoperative MRI and wrist arthroscopy was limited to 6 months. The exclusion criteria were as follows: previous surgical treatment for wrist joint diseases, wrist or forearm fracture history. Out of the 214 patients meeting the inclusion criteria, 21 were excluded due to the exclusion criteria.

Patients confirmed to have Palmer 1 (traumatic) TFCC injuries through wrist arthroscopy were included in this study. After arthroscopy, their preoperative MRI diagnoses were retrospectively retrieved for statistical analysis. Detailed information of 193 patients with traumatic TFCC tears are listed in Table 1.

**Table 1:** Demographic characteristics of 193 patients with traumatic TFCC tears

Demographic Characteristics	
Average age (years) (SD)	38 (12.2)
Gender, female (n) (%)	103 (53)
Gender, male (n) (%)	90 (47)
Mean duration from injury to surgery (months) (SD)	13 (14.7)
Mean duration from MRI to surgery (months) (SD)	2 (1.8)
Affected side, right (n) (%)	118 (61)
Affected side, left (n) (%)	75 (39)

### MRI

To capture the detailed and delicate structures of the TFCC, a high-field 3 Tesla MR scanner (GE MR750) was utilized to acquire high-resolution and high-contrast imaging data. Patients were placed in the “Superman position”, where the hand was raised above the head to ensure the wrist was positioned at the isocenter of the magnetic field for scanning purposes [19]. The wrists were scanned using an eight-channel wrist coil, which enhanced the clarity of high-resolution imaging and provided detailed visualization of the small structures of the TFCC. High-resolution proton density coronal sequences with both fat and non-fat suppression, sagittal T2-weighted fat-suppression sequences, and axial proton-density fat-suppression sequences were employed. Three orthogonal planes were obtained to ensure a good correlation between them. We only performed native MRI examinations and did not use contrast-enhanced examinations such as ceMRI or MR arthrography. Specific MRI settings can be found in Table 2.

**Table 2:** MRI sequence parameters

Sequence	FoV (mm)	Slice thickness /Interslice gap (mm)	Resolution	TR (ms)	TE (s)	Time (min)
Ax T2 FSE	10	2 mm/0 mm	320*256	2923	110	2.5
OCor 3D MRGE T2	10	0.6 mm/0 mm	300*300	linimum	18	6
Cor CUBE T2	10	1 mm/0 mm	256*224	1500	aximum	4
OSag CUBE T2	10	1 mm/0 mm	256*224	1500	aximum	3.5
Radial T2 GRE	12	1 mm/0.2 mm	256*224	440	15	5

FoV, field of view; TR, repetition time; TE, echo time

**Fig. 1:**
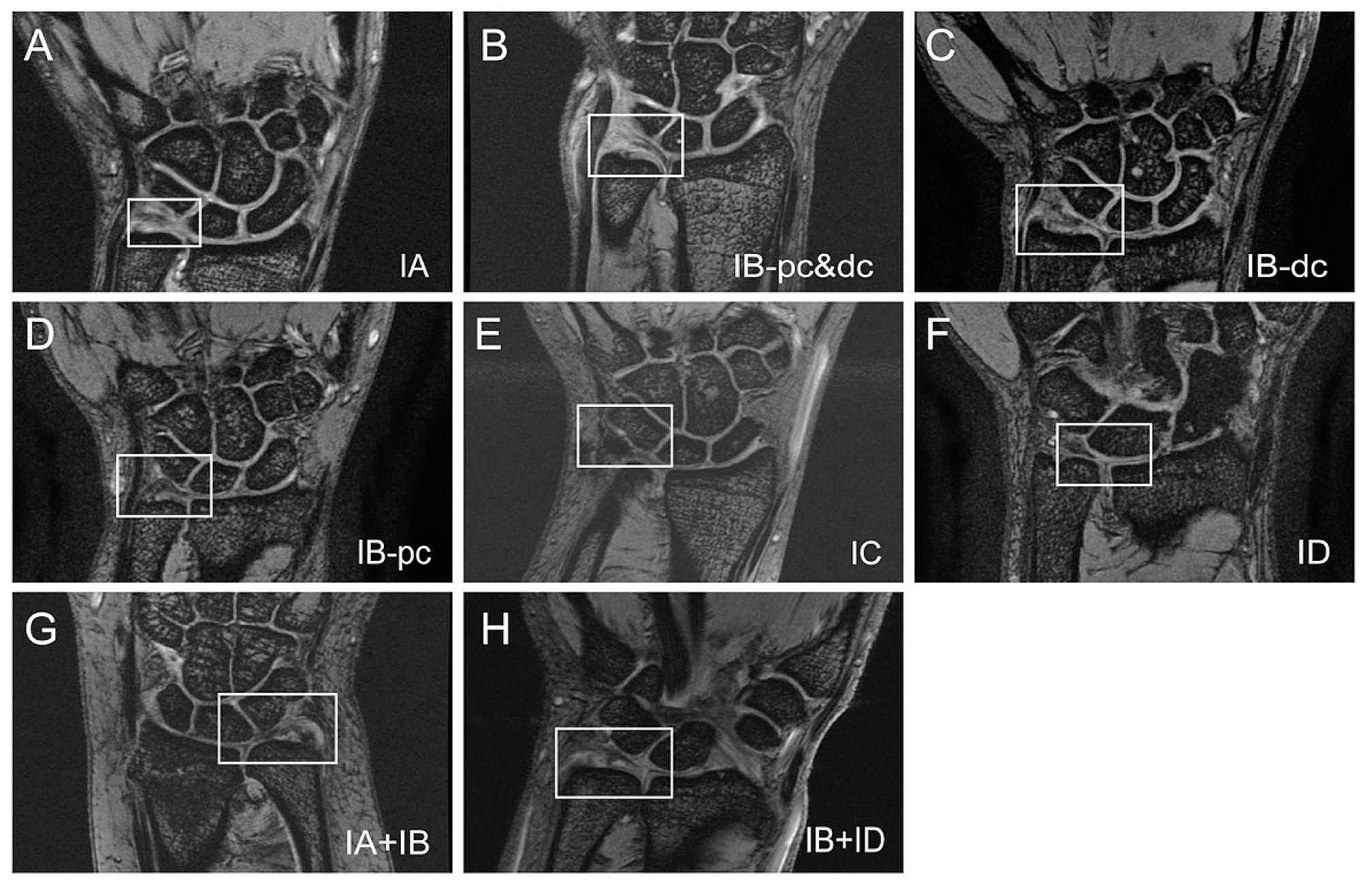
MRI images of the traumatic TFCC injuries according to Palmer’s classification. pc, proximal component; dc, distal component; MRI, magnetic resonance imaging; TFCC, triangular fibrocartilage complex

MRI of the injured wrists were examined by two radiologists from our center, and they both assessed all 193 patients. The interrater correlation (ICC) between the assessments of two radiologists was 0.97. MRI manifestations of traumatic TFCC injuries were shown in Fig. 1.

### Wrist arthroscopy

All 193 patients underwent wrist arthroscopy by three hand surgeons at our center. To preserve the integrity of the study and ensure unbiased arthroscopic evaluations, all hand surgeons involved in the wrist arthroscopy procedures could view the MRI images before the surgery, but they were blinded to the MRI diagnoses made by radiologists, especially the specific classifications. This procedure ensured that all the surgeons’ intraoperative observations and judgments were not influenced by prior knowledge of the MRI diagnoses.

Comprehensive surgical examination was conducted on all wrist joints, including the distal radioulnar joint, radiocarpal joint, and midcarpal joint. All arthroscopically observed ligament injuries were recorded, including TFCC injuries, lunotriquetral ligament injuries, and scapholunate ligament injuries. The subtypes of TFCC injuries were initially diagnosed by the operating surgeon during wrist arthroscopy and then reconfirmed by two other hand surgeons by reviewing surgical video records to ensure inter-observer reliability, according to Palmer’s and Atzei’s classification. These two reviewers who assessed the arthroscopy videos were blinded to the operating surgeons’ diagnoses. The ICC between these three hand surgeons was 0.95.

As extensively detailed by Aztei [3], TFCC injuries were observed through radiocarpal arthroscopy and assessed using the 6-R portal. The tension of TFCC was evaluated through the trampoline test and the hook test using the 4–5 or 6-R portal. Distal radioulnar joint arthroscopy was also performed because it remains the best method for identifying ligamentous tears of the pc-TFCC or avulsion of its foveal attachment. This procedure is deemed necessary when a positive hook test is observed and/or when the TFCC tear is associated with DRUJ instability. To assess the pc-TFCC, an 18-gauge hypodermic needle was employed to probe the foveal insertion. To assess the midcarpal joint, both the midcarpal radial portal and the midcarpal ulnar portal were used.

### Statistical analysis

The MRI findings were compared to the wrist arthroscopic findings, and the sensitivity, specificity and accuracy were calculated. In order to interpret the overall agreement, the investigators adhered to the criteria published by Landis and Koch [20], the comparison of proportions and agreement analysis was evaluated by Chi-square test and Cohen’s kappa [21]. Kappa values between 0.81 and 1.00 indicate almost perfect strength of agreement; 0.61–0.80 indicate substantial; 0.41–0.60 indicate moderate; 0.21–0.40 indicate fair. Sensitivity, specificity, positive and negative predictive values, and accuracy (defined as the sum of concordant cells divided by the sum of all cells in the 2-by-2 table) [22], along with their corresponding 95% confidence intervals, were calculated from the standard statistical tables. Among these evaluation indicators, negative predictive value (NPV) was particularly emphasized as it defines how many cases of TFCC injury were missed during MRI diagnostics.

## Results

### Proportion of different types of traumatic TFCC tears verified by arthroscopy

According to wrist arthroscopy in our center, traumatic TFCC injuries were subtyped into four types according to Palmer’s classification and two kinds of mixed types (1 A + 1B and 1B + 1D). The detailed proportion of different TFCC injuries can be found in Table 3.

**Table 3:** Proportion of different subtypes of traumatic TFCC injuries in arthroscopy (Palmer’s classification)

Palmer’s classification	No. (%), n = 193
1 A	14 (7)
1B	131 (68)
1 C	1 (1)
1D	4 (2)
1 A + 1B	27 (14)
1B + 1D	16 (8)

### Ratio of different types of TFCC peripheral tears verified by arthroscopy

Peripheral TFCC tears (Palmer 1B) can be further classified into four classes, according to Atzei’s classification. In this study, the investigators analyzed the proportion of each class of Atzei’s classification in 174 patients with peripheral TFCC tears (including type Palmer 1B, 1 A + 1B, 1B + 1D). The remaining 19 patients without Palmer 1B TFCC injuries, who were classified as Palmer 1 A, 1 C, or 1D, were not included in this section. The detailed proportion was shown in Table 4.

**Table 4:** Proportion of different types of TFCC peripheral tears in arthroscopy (Atzei’s classification)

Atzei’s classification	Styloid lamina (Distal component)	Foveal lamina (Proximal component)	DRUJ instability	No. (%), n = 174
Class 1 Distal tear	Tear	Intact	No	102 (59)
Class 2 Foveal avulsion	Intact	Tear	Yes	17 (10)
Class 3 Complete tear	Tear	Tear	Yes	52 (30)
Class 4 Massive rupture irreparable	Tear	Tear	Yes	3 (2)

### Diagnostic value of MRI in the qualitative and localization diagnosis of TFCC tears compared with arthroscopy

The diagnostic results of wrist arthroscopy were used as the “gold standard”. NPV, the most important evaluation indicator, reached up to 0.97 in traumatic TFCC injuries, indicating the extremely high diagnostic value of MRI. The accuracy for dc-TFCC and pc-TFCC was only moderate to fair. The detailed diagnostic value of MRI is shown in Table 5.

**Table 5:** Diagnostic value of MRI in the diagnosis of TFCC tears compared with arthroscopy

Types	Sensitivity	Specificity	PPV	NPV	Accuracy	Kappa Value	Agreement Degree
Traumatic	0.99	0.90	0.98	0.97	0.98	0.93	Almost perfect
	0.97-1	0.78-0.96	0.95-0.99	0.87-1	0.95-0.99		
dc-TFCC	0.93	0.53	0.95	0.47	0.89	0.44	Moderate
	0.88-0.96	0.30-0.75	0.9-0.97	0.26-0.69	0.83-0.93		
pc-TFCC	0.85	0.38	0.49	0.79	0.57	0.21	Fair
	0.74-0.92	0.29-0.49	0.39-0.58	0.65-0.89	0.49-0.65		

Abbreviations: PPV, positive predictive value; NPV, negative predictive value; pc, proximal component; dc, distal component; TFCC, triangular fibrocartilage complex

### Occurrence rates of associated injuries of TFCC tears detected by arthroscopy and radiological examination

Apart from directly observable tears of the TFCC, some associated injuries of wrist joint found in arthroscopy and radiological examinations, such as lunate and triangular cysts, may also be conducive to diagnose TFCC injuries. The epicenter method [23] was used in CT evaluation to ascertain the ulna’s position relative to the radius, and to assess DRUJ instability [24]. In this study, we selected several of the most frequently occurring associated injuries, and their occurrence rates are presented in Table 6.

**Table 6:** Occurrence rates of associated injuries of TFCC tears

Type	No. (%)	Surgical probe	MRI		CT
		Lunotriquetral injury	Lunate cysts	Triangular cysts	DRUJ instability
IA	14 (7)	4 (29)	5 (36)	4 (29)	5 (36)
IB	131 (68)	17 (13)	28 (21)	31 (24)	44 (34)
IC	1 (1)	0	0	0	1 (100)
ID	4 (2)	0	2 (50)	1 (25)	2 (50)
IA + IB	27 (14)	6 (22)	4 (15)	4 (15)	10 (37)
IB + ID	16 (8)	5 (31)	6 (38)	6 (38)	8 (50)
Total	193	32 (17)	45 (23)	46 (24)	70 (36)

## Discussion

This study evaluated the diagnostic value of MRI for TFCC injuries in comparison to wrist arthroscopy, and reported the proportions of various subtypes of TFCC injuries in regular clinical practice using a large sample dataset. The most common TFCC injury subtype was peripheral injury (Palmer 1B), accounting for 67.9% of cases. This was followed by combined injuries, with 14% classified as Palmer 1 A + 1B and 8.3% as Palmer 1B + 1D. MRI has shown almost perfect diagnostic value in traumatic TFCC injuries compared to wrist arthroscopic evaluation. However, it has limitations in providing detailed classification of TFCC tears.

Certain atypical TFCC injuries do not fit into Palmer’s classification, and combined injuries, which are frequently encountered in clinical practice, are also not accounted for in Palmer’s classification. Abe et al. reported a prevalence of 18.5% (32 out of 173 wrists) for combined tears, but specific subtypes of these combined tears were not specified [1]. In this study, we identified two primary types of combined TFCC injuries: those involving a combination of peripheral tear and central / radial TFC disc tear, predominantly categorized as Palmer 1B + 1 A and 1B + 1D. In such scenarios, an optimized management strategy should be devised to address TFCC injuries, with particular attention given to tears located at the distal radioulnar joint (DRUJ).

Following the precise elucidation of the anatomical structure of the TFCC [2] and advancements in surgical techniques for wrist arthroscopy, Atzei introduced a treatment-oriented classification of peripheral tears of the TFCC in 2009 [3]. In this study, distal tears (58.6%) and complete tears (involving both the distal and proximal regions, 29.9%) were relatively common in peripheral TFCC tears (Table 4). In wrist arthroscopy, arthroscopic-assisted TFCC foveal reattachment is performed through a dedicated DRUJ working portal, known as the direct foveal portal. Furthermore, the management of combined proximal and distal TFCC tears with TFC disc central and radial tears (Palmer 1 A + 1B and 1B + 1D) could be considered as an addition to Atzei’s classification due to their high prevalence of DRUJ instability, with proportions of 37% and 50%, respectively (Table 5). Simple dc-TFCC tear (Atzei Class 1) can be repaired by directly suturing of the injury. However, in cases where pc-TFCC tear (Atzei Class 2/3) associated with DRUJ instability is present, more complex surgical procedures are required to ensure TFCC reattachment to the fovea. Therefore, the diagnostic ability to localize the TFCC tear preoperatively would provide valuable guidance for developing an operative plan.

As suggested in the interdisciplinary consensus statements proposed by Luis Cerezal et al. [24], MRI is considered the most valuable technique for diagnosing TFCC injuries. In our study, there was almost perfect agreement between MRI and wrist arthroscopic examination in detecting traumatic TFCC injuries. Although, for localized TFCC peripheral tears, the agreement was moderate for dc-TFCC tears and fair for pc-TFCC tears (Table 5). This indicates that MRI is effective in detecting TFCC injuries but may not precisely determine the location of peripheral tears, particularly in the pc-TFCC tears. These findings align with results reported in other MRI studies pertaining to TFCC injuries, the most challenging aspect of detecting TFCC tears is identifying peripheral abnormalities such as tears at the ulnar side, the ulnar styloid, and foveal attachments [25, 26].

The Atzei classification we performed was based on direct observation during wrist arthroscopy, allowing us to clearly determine whether it’s a proximal or distal tear. However, when diagnosing TFCC injuries solely through MRI images, it may not provide the fine-grained details required for specific subtyping, which is important in clinical decision-making and treatment planning. Factors such as anatomical complexity, limitations in imaging techniques, variations in injury size, and local inflammation contribute to the challenges in accurately identifying these specific injury subtypes via MRI. In such circumstances, MR arthrography and CT play a crucial role as complementary examinations for diagnostic classification, as they can provide essential insights into structural abnormalities and potential ligamentous injuries that support the clinical diagnosis of DRUJ instability [27–29]. By MR arthrography, the rupture ends of the foveal and styloid lamina are distended, and thus IB ruptures according to Palmer become visible at all [30]. CT is the most useful and accurate imaging technique for assessing DRUJ instability, as it directly evaluates the dorsal subluxation of the ulnar head [24]. Moreover, in contrast-enhanced MRI, intravenous gadolinium contrast agent can facilitate higher diagnostic accuracy and confidence the detection of fresh lamina ruptures of the TFCC due to focal hypervasularization [31].

This study has some limitations due to its retrospective design and single-center setting. If it had included different clinical practitioners, institutions, and countries, the research results might have resulted in variation of the research findings. Moreover, the study only focused on patients with traumatic TFCC injuries, thereby excluding degenerative injuries, which could potentially restrict the generalizability of our findings. MRI has limited diagnostic value in further localizing tears in peripheral TFCC injuries, requiring other diagnostic methods to address this challenge. Regarding associated injuries such as lunotriquetral dissociation, we only reported their occurrence rates among different Palmer types and did not analyze their correlation. In the future, more targeted clinical studies will be needed to determine their specific diagnostic value in TFCC injuries.

Although MRI remains the most useful technique for diagnosing TFCC injuries, wrist arthroscopy, performed after a comprehensive clinical examination, remains the most effective procedure for the assessment and treatment of TFCC injuries in a contemporary practice. Furthermore, wrist arthroscopy is the only diagnostic tool that dynamically can assess the grade of instability and the healing capacity of the injury.

## Conclusions

The diagnostic value of MRI in traumatic TFCC injuries has been confirmed to be almost perfect using Palmer’s classification. In more detailed classification of TFCC injuries, such as pc-TFCC tears classified by Atzei’s classification, the diagnostic accuracy of MRI remains lower compared to wrist arthroscopy. Radiological associated injuries may offer additional diagnostic value in cases with diagnostic uncertainty.

## Data Availability

The datasets used and/or analysed during the current study are available from the corresponding author on reasonable request.

## Abbreviations

dc-TFCC: Distal component of TFCC
DRUJ: Distal radioulnar joint
ICC: Interrater correlation
MRI: Magnetic resonance imaging
NPV: Negative predictive value
pc-TFCC: Proximal component of TFCC
PPV: Positive predictive value
TFCC: Triangular fibrocartilage complex
